# Normalization of drug and therapeutic concepts with Thera-Py

**DOI:** 10.1093/jamiaopen/ooad093

**Published:** 2023-11-08

**Authors:** Matthew Cannon, James Stevenson, Kori Kuzma, Susanna Kiwala, Jeremy L Warner, Obi L Griffith, Malachi Griffith, Alex H Wagner

**Affiliations:** The Steve and Cindy Rasmussen Institute for Genomic Medicine, Nationwide Children’s Hospital, Columbus, OH, United States; The Steve and Cindy Rasmussen Institute for Genomic Medicine, Nationwide Children’s Hospital, Columbus, OH, United States; The Steve and Cindy Rasmussen Institute for Genomic Medicine, Nationwide Children’s Hospital, Columbus, OH, United States; Division of Oncology, Department of Medicine, Washington University School of Medicine, St. Louis, MO, United States; Department of Medicine, Brown University, Providence, RI, United States; Division of Oncology, Department of Medicine, Washington University School of Medicine, St. Louis, MO, United States; Division of Oncology, Department of Medicine, Washington University School of Medicine, St. Louis, MO, United States; The Steve and Cindy Rasmussen Institute for Genomic Medicine, Nationwide Children’s Hospital, Columbus, OH, United States; Department of Pediatrics, The Ohio State University College of Medicine, Columbus, OH, United States

**Keywords:** therapeutics, medical informatics, biological ontologies, knowledge bases, health information interoperability

## Abstract

**Objective:**

The diversity of nomenclature and naming strategies makes therapeutic terminology difficult to manage and harmonize. As the number and complexity of available therapeutic ontologies continues to increase, the need for harmonized cross-resource mappings is becoming increasingly apparent. This study creates harmonized concept mappings that enable the linking together of like-concepts despite source-dependent differences in data structure or semantic representation.

**Materials and Methods:**

For this study, we created Thera-Py, a Python package and web API that constructs searchable concepts for drugs and therapeutic terminologies using 9 public resources and thesauri. By using a directed graph approach, Thera-Py captures commonly used aliases, trade names, annotations, and associations for any given therapeutic and combines them under a single concept record.

**Results:**

We highlight the creation of 16 069 unique merged therapeutic concepts from 9 distinct sources using Thera-Py and observe an increase in overlap of therapeutic concepts in 2 or more knowledge bases after harmonization using Thera-Py (9.8%-41.8%).

**Conclusion:**

We observe that Thera-Py tends to normalize therapeutic concepts to their underlying active ingredients (excluding nondrug therapeutics, eg, radiation therapy, biologics), and unifies all available descriptors regardless of ontological origin.

## Background and significance

Harmonizing all existing names for any one given therapeutic concept has been a challenging problem for medical informatics in recent decades.^1–3^ In modern medical practice, medical professionals are frequently expected to synthesize therapeutic knowledge of drug mechanisms, effectiveness, and other metrics to design treatment regimens that achieve the best possible outcomes for their patients. Databases and other resources exist that allow medical professionals to collect information about a therapeutic, but this process can be hampered due to the ambiguity associated with therapeutic naming strategies. Harmonizing even a single therapeutic requires curated knowledge of all possible identifiers of active ingredients, chemical structures, developmental aliases, and generic or brand names.^4^ This problem is exacerbated in clinical genomics, where ambiguity (or a lack of standardization) can confound treatment decision-making.

Consider imatinib, a tyrosine kinase inhibitor that was first used to treat Philadelphia chromosome-associated chronic myelogenous leukemia.^5^ This same drug was initially marketed as Gleevec in the United States and Glivec in the EU, by the Swiss-American pharmaceutical company Novartis; additional generic brand names now include Celonib, Enliven, Gleevac, Imalek, Imatib, Mesylonib, Mitinab, Plivatinib, Shantinib, Temsan, and Veenat. Before any of these brand names were assigned to the therapeutic, it was published under the identifier “STI-571” in the medical literature.^6^ It can additionally be referenced by the different salt formulations present on the market (imatinib mesylate or imatinib methanesulfonate), or by its chemical structure:

$$\begin{array}{c} \alpha -\left(4-\text{methyl}-1-\text{piperazinyl}\right)-3\prime \\ -\left(\left(4-\left(3-\text{pyridyl}\right)-2-\text{pyrimidinyl}\right)\text{amino}\right)\\ -p-\text{tolu}-p-\text{toluidide} \end{array}$$

Despite their different ontological origins, the preceding examples are all contextually equivalent when referenced with respect to drug–gene or drug–variant interaction annotations, even if there may be subtle distinctions in other, nontherapeutic contexts. Standards and naming conventions exist at every level of development, from internal pharmaceutical development compound identifiers (eg, AZD-####) and chemical structure names employed in early development pipelines, to fully-realized brand and marketing names with myriad formulations defined by subgroups of additives and delivery mechanisms.^7^ This notion has driven regulatory bodies and programs (such as the United States Adopted Names program) to assign generic names reflecting the underlying active ingredients prior to their marketing. Changes such as these were made in an effort to unify ambiguously named products and protect consumers.^8^ Thus, no matter the stage of development, all assigned names have some tangible link to one another through their relation as a descriptor to the underlying active ingredient(s).

To bridge therapeutic ambiguity, we introduce Thera-Py, a Python package and web API that constructs searchable merged concepts for drugs and therapeutic terminologies using publicly available therapeutic resources and thesauri. Merged concepts are constructed from an aggregate set of traits, trade names, and aliases that act as a cross-resource mapping to enable more refined data processing for downstream clinical and research applications. In this report, we outline the methodology behind Thera-Py and provide an analysis on normalization rates across different data sources. Further, we examine the challenges of normalization of therapeutic terminologies and provide suggestions on improving data standards to support improving data harmonization.

## Results

### Normalization/grouping routine

**Figure 1.**
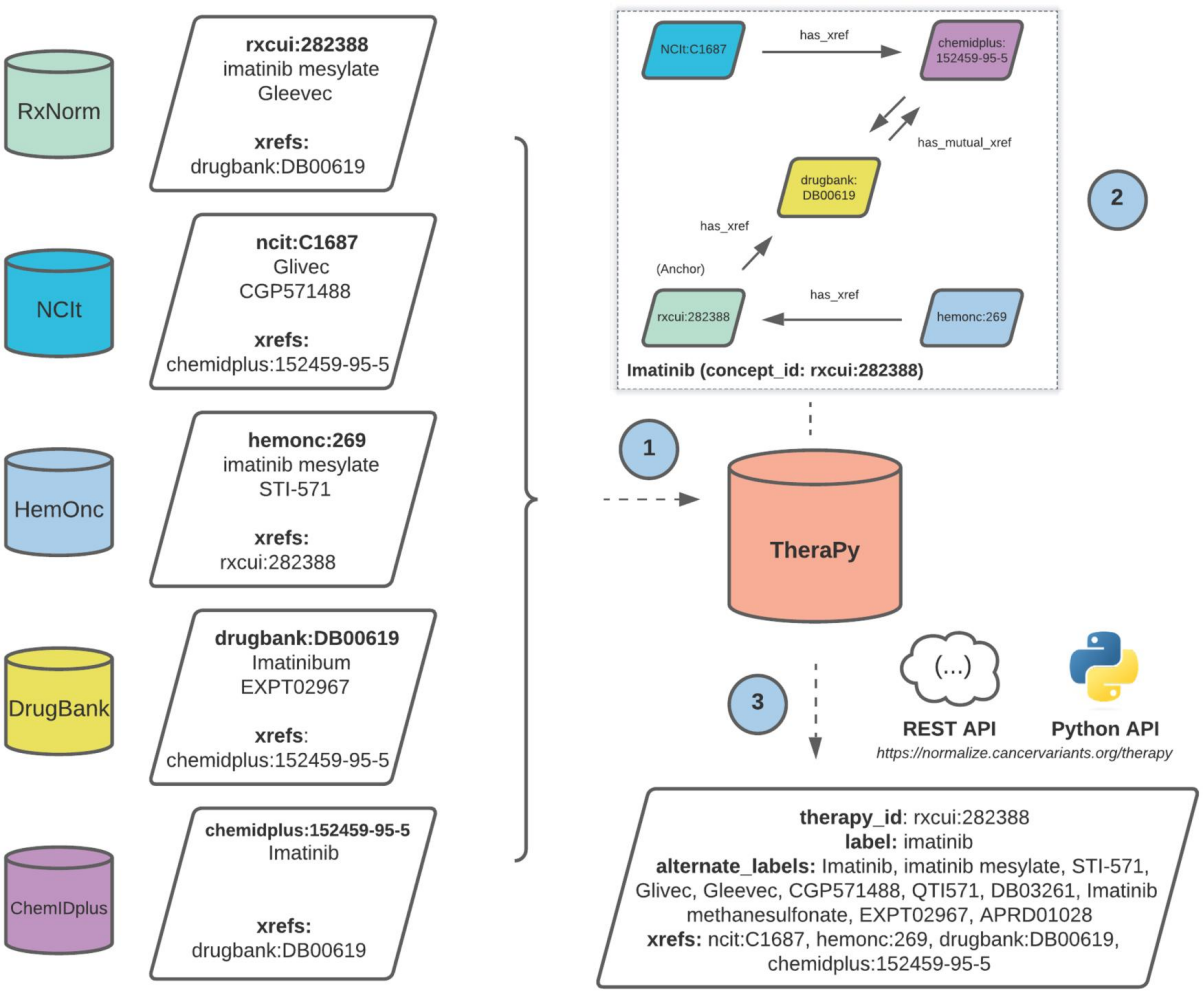
Normalization of therapeutic concepts using Thera-Py. Schematic workflow of concept normalization methodology. Shown above is an example workflow for import and normalization of records relating to Imatinib. (1) Therapeutic records are imported from aggregate sources and separated into representative points for all aliases, trade names, explicit cross-references (xrefs), as well as other associations and descriptors. (2) Cross-references are used to draw “has reference to” relationships to similar therapeutics across other sources to create networked groups. The starting node used to create the network is considered the anchor node and becomes the reference identifier for the therapeutic concept. Starting nodes are initialized from an internal source priority hierarchy whereby sources designed for clinical decision-making through expert curation were given higher priority than generalized sources. (3) All networked groups are linked under one merged concept record. Raw therapeutic inputs can be normalized to their merged concept record via Thera-Py for downstream clinical and research applications. Available from: https://normalize.cancervariants.org/therapy/.

Thera-Py utilizes community-driven vocabularies to generate stable concept mappings between identifiers (Figure 1). We aggregated concept codes from 9 therapeutic ontologies and vocabularies. Terms were extracted from: Wikidata,^9^ HemOnc,^10^ ChEMBL,^11^ the National Cancer Institute Thesaurus^12^ (NCIt), RxNorm,^13^ ChemIDplus,^14^ Drugs@FDA,^15^ DrugBank,^16^ and the IUPHAR Guide to Pharmacology.^17^ These sources were chosen due to their high public use as well as the diversity of scope and knowledge contained within each sources. We then developed an algorithm to cross-map extracted concept codes and link together records. Normalized identity records are generated in a 2-step process:

1. Directed graphs are constructed from source data, where records from each source act as nodes and “has reference to” relationships act as edges between nodes. These relationships are explicit, curated references (xrefs) from one record to another (eg, the record rxcui: 282388 explicitly references drugbank: DB00619) (shown in Figure 1).
2. Each set of connected nodes is related as a distinct, unified therapeutic concept and assigned a common identifier. All aliases, trade names, annotations, associations, regulatory approvals, and indications are merged under this identifier.

Starting nodes were chosen according to an internal source trust ranking, where records with higher priority were used to initialize groups whenever possible. Sources were ordered according to their perceived therapeutic scope where those designed and annotated primarily for clinical decision-making (usually through expert curation) ranked higher than generalized sources. Thusly, the source priority order used for anchor node decision-making, from most preferred to least preferred, was: RxNorm, NCIt, HemOnc, Drugbank, Drugs@FDA, IUPHAR Guide to Pharmacology, ChEMBL, ChemIDplus, followed by Wikidata.

### Creation and access of normalized concepts

We ran our normalization routine via Thera-Py as described previously in the Methods section (also available from https://go.osu.edu/TPY). Distinct sets of nodes were assigned a stable merged concept identifier with all associated aliases, trade names, and other therapeutic descriptors associated (Figure 1). All merged therapeutic descriptors and cross-references remained accessible via their assigned stable concept identifier.

A total of 16 069 merged groups were created for different therapeutic concepts. These merged groups were assigned identifiers reflective of the anchor node (Figure 1) used to create each group: NCIt (6574 groups, 40.9%), RxNorm (4647 groups, 28.9%), GuideToPharmacology (3490 groups, 21.7%), DrugBank (1214 groups, 7.6%), ChEMBL (93 groups, 0.5%), ChemIDplus (51 groups, 0.3%) (Figure S1a). Of all merged concepts created, 84.7% of groups contained between 2 and 5 records (Figure S1b). The remaining 15.3% of groups contained anywhere from 6 to 86 records. The merged groups with the largest number of combined records are highlighted in Table S1.

### Analysis of concept normalization rates

**Figure 2.**
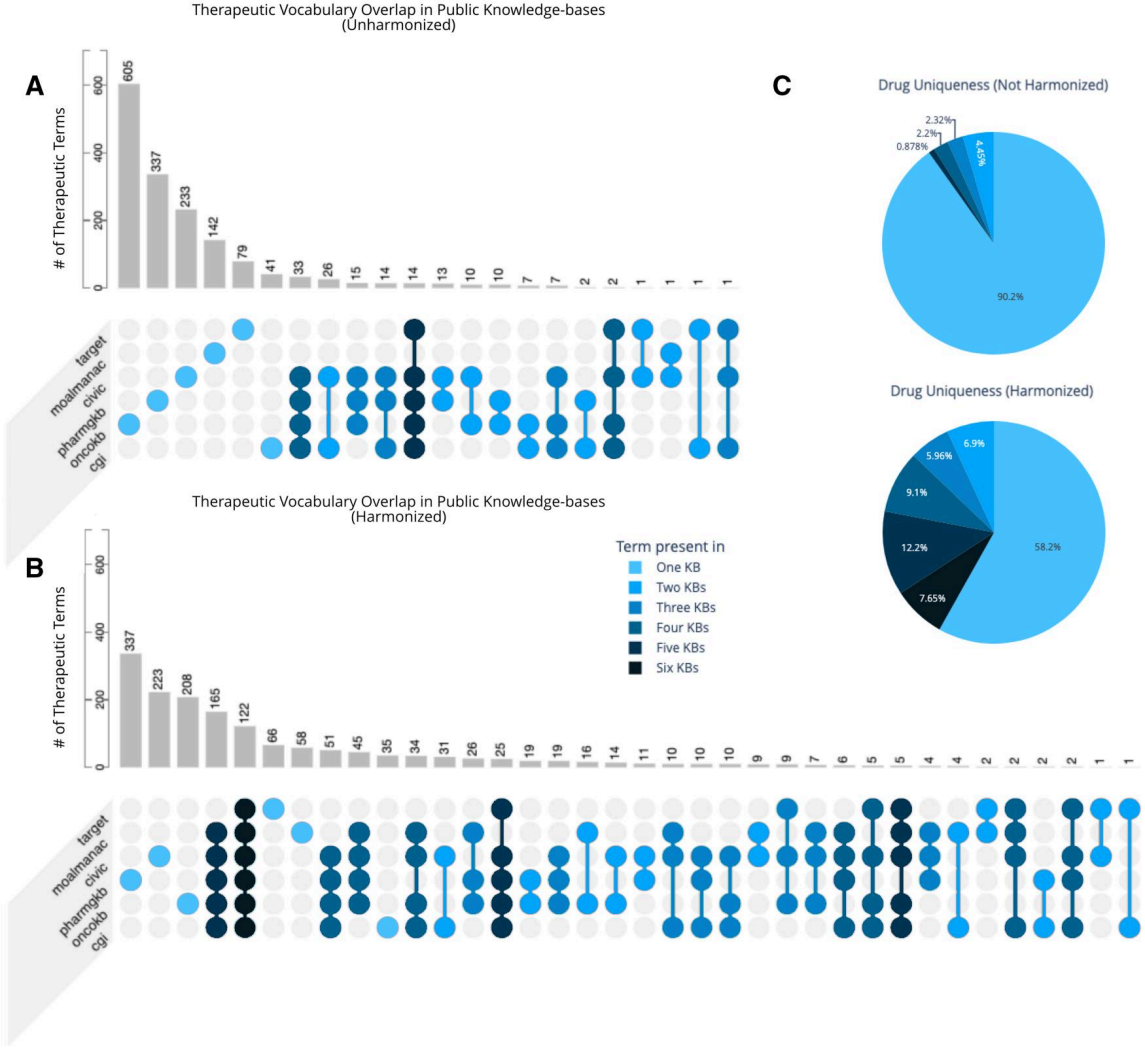
Intersection of therapeutic vocabulary from public knowledge-bases, pre-, and postharmonization using Thera-Py. Therapeutic terminology was obtained from 6 different publicly available drug vocabularies as a test set to evaluate cross-resource therapeutic overlap. (A) Test sets of therapeutic terminology were compared via string matching to quantify the number of exact matches present across resources. The intersections of resources with exact matches are highlighted and colorized by the number of contributing resources. (B) Test sets of therapeutic terminology were harmonized using Thera-Py and then compared via concept ID to evaluate the number of matches across resources. Terminologies with exact matches for their concept IDs (irrespective of their original vocabulary term) were quantified. The intersections of resources with matches are highlighted and colorized by the number of contributing resources. (C) Drug uniqueness of therapeutic vocabulary across resources pre- and postharmonization using Thera-Py. Uniqueness is quantified as the number of terms present in various knowledge-bases intersection sizes.

To evaluate the ability of Thera-Py to successfully harmonize therapeutic terminology across resources, we obtained searchable drug vocabularies from 7 different publically available knowledgebases to act as our test set. These knowledgebases are distinct from those used to build Thera-Py and comprised Memorial Sloan Kettering (MSK) Precision Oncology Knowledge Base (OncoKB),^18^ Pharmacogenomics Knowledgebase (PharmGKB),^19^ Clinical Interpretation of Variants in Cancer (CIViC),^20^ Cancer Genome Interpreter Cancer Biomarkers Database (CGI),^21^ Molecular Oncology Almanac (MOAlmanac),^22^ Tumor Alterations Relevant for Genomics-Driven Therapy (TARGET),^23^ and the Drug-Gene Interaction Database (DGIdb).^24^ Prior to normalization, therapeutic terminology was compared via string matching to obtain the intersection of common terminology across resources (Figure 2). DGIdb was not included in this preliminary analysis due to its nature as an aggregate resource. Our analysis showed a total of 1198 terms unique to a single resource, with 115 and 58 terms being shared across 4 and 5 resources, respectively.

**Figure 3.**
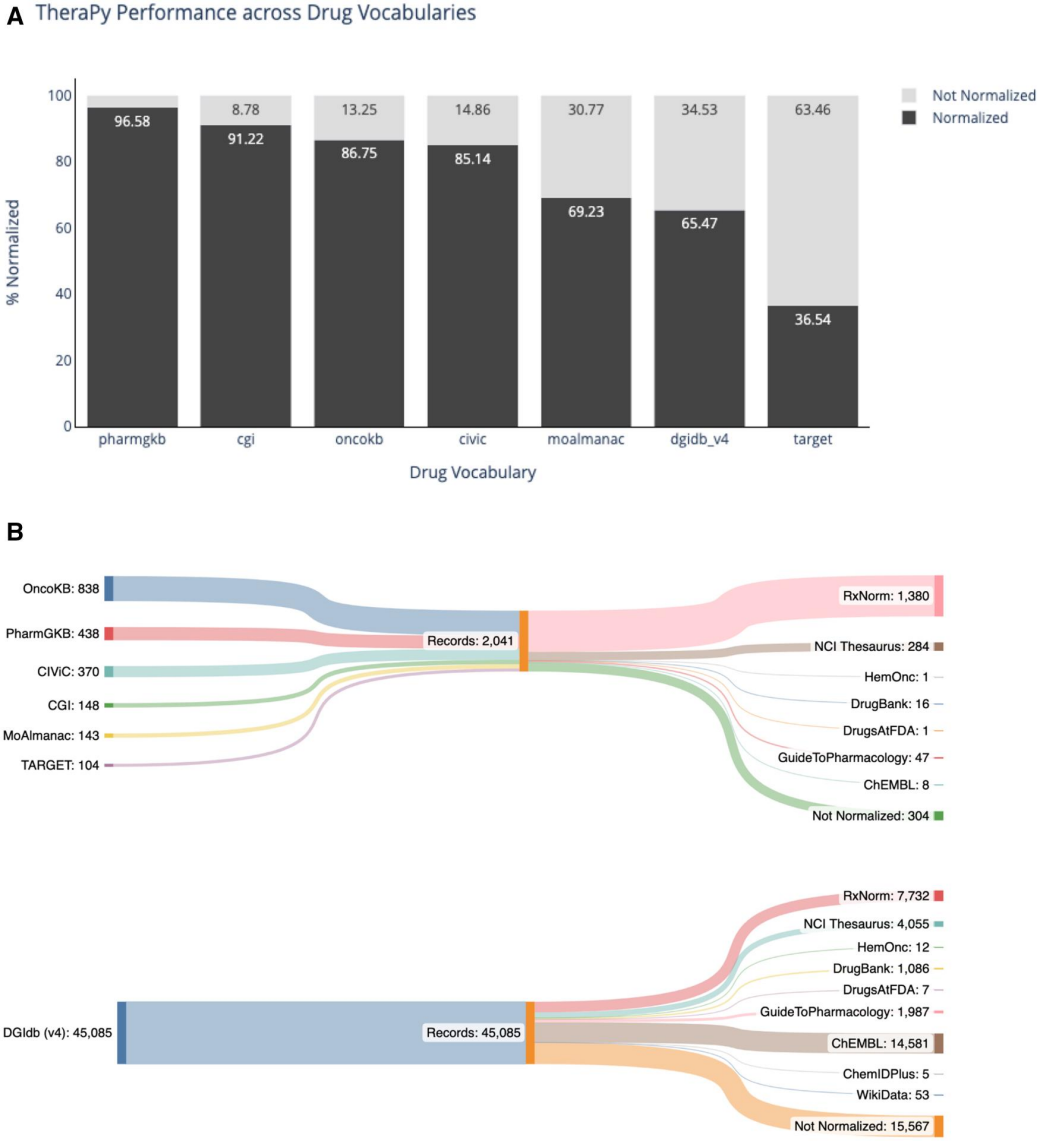
Thera-Py normalization performance using publicly available drug vocabularies. (A) Normalization performance for therapeutic terms obtained from 7 different publicly available resources. (B) Parent node representation for normalized therapeutic terms taken from different resources. Source priority is represented via verticality with records that failed to normalize at the bottom.

The unique set of terms from each source was then normalized using a local installation of Thera-Py (v.0.3.6) and merged concept identifiers were obtained for each term (Figure S2). The lack of a merged concept identifier for a unique term was deemed as a failure to normalize. This analysis showed high normalization rates for 4 of 7 sources: PharmGKB (95.6%), CGI (91.2%), OncoKB (86.7%), and CIVIC (85.1%) (Figure 3A). The remaining 3 sources saw lower rates of normalization: MOAlmanac (69.2%), DGIdb (65.4%), and TARGET (36.5%). Examples of terms that failed to normalize are highlighted in Tables 1 and 2. The anchor nodes for each successfully retrieved merged concept were also recorded. Our analysis of anchor distributions showed RxNorm to be the most frequently-occurring anchor node for drug terms within 6 out of 7 drug sets: OncoKB, PharmGKB, CIVIC, CGI, MOAlmanac, and TARGET (Figure 3B). In contrast, ChEMBL was the most frequently occurring anchor node for drug terms obtained from DGIdb.

**Table 1.** Normalization failure terminology from publicly available drug vocabularies.

Terminology	Occurrences
chemotherapy	6
carboplatin-taxol regimen	3
r3mab	3
car-t cells targeting mesothelin	3
anti-vegf	2
parp inhibitors	2
hdac inhibitors	2
src inhibitors	2
egfr inhibitors	2
opium derivatives and expectorants	2
oxymetazoline and tetracaine	2
antithrombotic agents	2
paracetamol, combinations excl. psycholeptics	2
vitamin b-complex with vitamin c	2
parathyroid hormones and analogues	2
high dose chemotherapy	2
flourouracil	2
pc regimen	2
car-t cells targeting muc1	2
cat regimen	2
sb3	2
carbo-tax regimen	2
liposomal doxorubicin	2
sgk1-inh	2
radiation ionizing radiotherapy	2

Most frequently occurring terms (top 25) taken from drug vocabularies that were not able to return matches from Thera-Py and thus failed to normalize.

**Table 2.** Additional examples of normalization failure terminology from publicly available drug vocabularies.

Terminology	Description
HDAC inhibitors	General Category
MEK Inhibitors	General Category
beta blocking agents, nonselective	General Category
Tyrosine kinase inhibitors	General Category
Pyrrollidine derivative 3	Compound Identifier
Carbamate derivative 3	Compound Identifier
Tetra-hydro-isoquinoline derivative 4	Compound Identifier
Benzene sulfonamide derivative 3	Compound Identifier
EVT-103	Exp. Compound Identifier
ADR-851	Exp. Compound Identifier
APN-201	Exp. Compound Identifier
Cysplatyna	Multi-language Label
Flourouracil	Misspelled Label
Vandetinib	Misspelled Label
Radiation Therapy	Therapeutic Description
CD19 CAR Gene Transduced T Lymphocytes	Therapeutic Description
Anti-PD-L1 CSR T Cells	Therapeutic Description
Long-acting erythropoietin conjugate	Therapeutic Description
Interferon-alpha lozenge	Therapeutic Description
coxsackievirus type a21	Therapeutic Description
249565746	Unknown Identifier

Select terms taken from drug vocabularies that were not able to return matches from Thera-Py and thus failed to normalize. Descriptions of type of drug terminology have been provided next to each term.

## Discussion

Therapeutic vocabularies from public sources were subjected to directed graph construction to construct stable merged concepts for all descriptors for any given therapeutic concept. Our results showed the construction of 16 069 unique therapeutic concept groups from our import set. We found that 84.7% of all merged concepts created with this methodology contained between 2 and 5 records per group (Figure S1c). The remaining 15.3% merged concepts contained >5 records per group with the largest 25 groupings shown in Table S1. The size of these larger groupings can likely be attributed to the contributions of Drugs@FDA. This resource was added to Thera-Py to capture more accurate notions of regulatory approval for therapeutic concept groups through association with all active Abbreviated New Drug Application (ANDA) and New Drug Application (NDA). In doing so, however, this has inflated some therapeutic groups to larger sizes as evidenced by the group “rxcui: 21245” containing 84 records (79 of which are ANDA/NDA application records).

Our analysis of publicly available drug vocabularies showed high rates of normalization for terms obtained from 5 of 7 sources, with TARGET and DGIdb seeing lower rates of normalization for vocabularies (30%, 65.3%, respectively). We expect the lower rates of normalization in these 2 sources to be likely due to more frequent occurrences of general categories (eg, HDAC Inhibitors, MEK Inhibitors), nonspecific identifiers (eg, Pyrrolidine derivative 3, Carbamate derivative 3), misspelled or uncaptured multilanguage labels (eg, “Vandetinib,” Cysplatyna), or unlisted experimental compound identifiers (eg, EVT-103, ADR-851). Additionally, some terms present within these datasets proved to be therapeutically descriptive but difficult to normalize (eg, CD19 CAR Gene Transduced T Lymphocytes, Anti-PD-L1 CSR T Cells, Long-acting erythropoietin conjugate). While Thera-Py does not support fuzzy checks or approximate string matching in its current form, these techniques could be implemented later to handle some of these difficult terminologies. Additionally, the recent development of large language model (LLM) based methodologies could potentially enhance our ability to handle difficult therapeutic terminologies.

We found that our methodology tended to favor normalizing therapeutic concepts to their active ingredient (as defined by USAN generic naming standards). Thera-Py was able to reliably capture relationships between the most used therapeutics at the level of generic names, brand names, and even developmental codes or chemical structures in some cases. Conversely, however, it was unable to capture broader therapeutic groupings such as “tyrosine kinase inhibitor” or “antibody therapy.” Using our approach, attempts to capture broader descriptors would lead to unintended downstream effects whereby all therapeutics would normalize to their broader therapeutic definition regardless of underlying ingredients (ie, erlotinib, dasatinib, or gefitinib all normalizing to “tyrosine kinase inhibitor”). The capture of these broader therapeutic classes likely has practical benefits for downstream applications, though their implementation would need to be defined to a different conceptual space within therapeutic concepts. For example, an additional field called “drug class” could be implemented that attaches groupings such as “tyrosine kinase inhibitor” to their relevant therapeutic concepts. This information could then enable harmonization of therapeutics from the point-of-view of drug classes as opposed to explicit underlying ingredients.

Interestingly, among the vocabularies used to create groups and subsequently test Thera-Py, we observed many different types of therapeutic categories all co-occurring within the same fields. These types included: natural products, chemical structures, development codes, generic names, brand names, product formulations, and treatment regimens. With all terms carrying a similar weight despite connotations of maturity, it is important to consider the nuances of what defines a “therapeutic” when applying a normalization strategy such as the one we introduced in Thera-Py.

Our results highlight a critical step for harmonizing therapeutic vocabularies in a computationally digestible format. By merging available records for any therapeutic concept, we are able to create a corresponding identifier that contains all aliases, trade names, and descriptors for commonly used therapeutics. These identifiers can be incorporated within bioinformatic and clinical workflows to unify therapeutic terminology regardless of origin, brand, or maturity stage. Merged records also have potential applications within machine learning workflows, where grouped descriptors can be used to aid in the generation of embeddings for downstream tasks.

More work remains to disambiguate the nuances between therapeutic concept domains and provide additional avenues for quality control of therapeutic concept groups. Future effort will require more precise encodings of semantic relations between classes, leveraging recent specifications such as SSSOM for unambiguous, standardized sharing of cross-domain concept mappings. We look forward to these developments, as success in this area will pave the way for applications such as Thera-Py to assist inference engines and the development of AI-driven clinical decision support capable of relating disparate therapeutic knowledge resources.

## Materials and methods

### Extraction of therapeutic concepts from resources

Records for drugs, therapeutics, and chemicals were obtained from individual publicly available resources: Terms were extracted from: Wikidata,^9^ HemOnc,^10^ ChEMBL,^11^ the National Cancer Institute Thesaurus,^12^ RxNorm,^13^ ChemIDplus,^14^ Drugs@FDA,^15^ DrugBank,^16^ and the IUPHAR Guide to Pharmacology.^17^ Further detail on extraction from each individual source can be found within Supplementary Methods. Records were imported directly as identity records and stored in a locally deployed DynamoDB instance. For each record, aliases, trace names, and database cross-references were extracted and stored as pointers to their original identity. Records within the DynamoDB instance are updated from parent knowledge bases on a quarterly basis.

### Analysis of normalization success rates

Drug terminology sets were obtained from 7 different publically available resources: the Memorial Sloan Kettering (MSK) Precision Oncology Knowledge Base (OncoKB),^18^ Pharmacogenomics Knowledgebase (PharmGKB),^19^ Clinical Interpretation of Variants in Cancer (CIVIC),^20^ Cancer Genome Interpreter Cancer Biomarkers Database (CGI),^21^ Molecular Oncology Almanac (MOAlmanac),^22^ Tumor Alterations Relevant for Genomics-Driven Therapy (TARGET),^23^ and the Drug-Gene Interaction Database (DGIdb).^24^ All drug terms from each source were normalized using a local installation of Thera-Py (v.0.3.6). Successful normalization was determined by the retrieval of a merged concept for each term. If a merged concept was not identified, that term was recorded as a failure of normalization.

## Supplementary Material

ooad093_Supplementary_Data

## Data Availability

Thera-Py is an open-source python package and is available for download and use at: https://github.com/cancervariants/therapy-normalization. An interactive demo is available from: https://normalize.cancervariants.org/therapy. Individual terms can be searched on this page to allow for manual inspection of therapeutic concepts without the need for locally hosted software. All therapeutic concepts were constructed from publicly accessible knowledge bases and are available for access via Thera-Py endpoints. The DynamoDB instance supporting Thera-Py is updated on a quarterly basis.
