# Supplementary material for: Normalization of drug and therapeutic concepts with Thera-Py
Source: JAMIA Open. 2023 Nov 8;6(4):ooad093. doi: 10.1093/jamiaopen/ooad093 (PMC10637840; doi:10.1093/jamiaopen/ooad093)
Supplement: ooad093_Supplementary_Data [file ooad093_supplementary_data.zip › Supplemental Methods.docx]

**SUPPLEMENTAL MATERIALS AND METHODS**

*HTML Access of Normalized Concepts*

Thera-Py concepts are accessible via HTTP requests to search across three endpoints: “search”, “normalize”, and “normalize_unmerged”, In all search types, Thera-Py compares the input term with all available concept identifiers, labels, trade names, aliases, cross-references, and associations to find case-insensitive string matches. Matches for the input term are returned in each endpoint and assigned a score corresponding to the type of match identified (Concept ID: 100, Label/Trade Name: 80, Alias/Xref/Association: 60, No Match: 0). Further documentation for these endpoints and their associated schemas can be found within our SwaggerUI documentation (<https://normalize.cancervariants.org/therapy>). Individual therapeutic terms can be input on this page to allow for manual exploration of therapeutic concepts without the need for locally hosted software.

*Python Access of Normalized Concepts*

Thera-Py is also available as a Python library, installable from the Python Package Interface (PyPI, https://pypi.org/project/therap-py/), and can be run on the command-line. In conjunction with a user-provided DynamoDB database instance (either installed locally or available via Amazon Web Services), data can be loaded by executing the command-line interface module with `--update_all` and `--update_merged` flags. This process will attempt to fetch the latest versions of all source data, extract relevant identifiers, terms, attributes, and cross-references, produce aggregate, normalized terms, and store finished records in the DynamoDB key-value table. Once data loading is complete, Thera-Py can be run and queried on a local machine. The project GitHub repository provides additional documentation and basic support for troubleshooting at <http://go.osu.edu/TPY>.

*Extraction of Therapeutic Concepts from Individual resources*

This section details further how data was imported directly from each individual source.

*ChEMBL* is a “large, open-access drug discovery database that aims to capture Medicinal Chemistry data and knowledge across the pharmaceutical research and development process”[^13^](https://paperpile.com/c/0sK7Ud/lwiE), developed by the European Bioinformatics Institute at the European Molecular Biology Laboratory (EMBL-EBI). Thera-Py ingests chemical identifiers, labels, aliases, and trade names. Additionally, structured regulatory approval information, including the “max phase” of approval from a survey of regulatory bodies, indicated diseases and phenotypes for use, and withdrawal status if relevant, is acquired. Data is extracted from the latest compressed sqlite distribution (chembl_XX_sqlite.tar.gz) available on the EMBL-EBI FTP server (<https://ftp.ebi.ac.uk/pub/databases/chembl/ChEMBLdb/latest/>).

*ChemIDplus* was a database storing molecular data about drugs, mechanisms of actions, drug interactions, and drug targets. Thera-Py retrieves CAS identifiers, aliases, and cross-references from the most recent ChemIDplus data dump (currentchemid.zip) available on the National Library of Medicine’s FTP server (ftp://[ftp.nlm.nih.gov/nlmdata/.chemidlease/](http://ftp.nlm.nih.gov/nlmdata/.chemidlease/)).

*DrugBank* is a “web-enabled database containing comprehensive molecular information about drugs, their mechanisms, their interactions and their targets.”[^24^](https://paperpile.com/c/0sK7Ud/lffC) Thera-Py extracts drug identifiers, labels, aliases, and cross-references from all the records in the CC0 dataset provided by DrugBank (drugbank vocabulary.csv) in their open data section (<https://go.drugbank.com/releases/5-1-10#open-data>).

*Drugs@FDA* contains FDA-approved labeling and ingredient information for prescription and over-the-counter drugs, as well as many therapeutic biologics. Thera-Py extracts ANDA and NDA marketing application numbers as concept identifiers, as well as trade names, aliases, and some cross-references based on active ingredients. Additionally, the product’s marketing status (discontinued, prescription, over-the-counter, or none) is stored. The Drugs@FDA dataset (drug-drugsfda-0001-of-0001.json.zip) is retrieved from the OpenFDA downloads page (<https://open.fda.gov/apis/drug/drugsfda/download/>).

*IUPHAR Guide to Pharmacology* is an “open-access, expert-curated database of molecular interactions between ligands and their targets”.[^25^](https://paperpile.com/c/0sK7Ud/xAhL) Thera-Py makes use of two GtoPdb files: the complete ligand list (ligands.csv) and the ligand ID mapping file (ligand_id_mapping.csv), both available on the GtoPdb downloads page (<https://www.guidetopharmacology.org/download.jsp>). For each ligand, concept identifiers, labels, aliases, cross-references, and approval data are pulled from the ligand list, and additional cross-references are extracted from the ligand ID mapping file. Approval data reflects whether the drug in question has been permitted for clinical use by at least one of a selection of regulatory agencies, or whether it has been removed from the market due to safety or other issues.

*HemOnc* is a freely available wiki providing information on therapeutics and protocols in hematology and oncology. A subset of this information is incorporated in the Observational Outcomes Partnership (OMOP) common data model (CDM), enabling its use in “systematic analysis of disparate observational databases… using a library of standard analytic routines and analytic tools”.[^20^](https://paperpile.com/c/0sK7Ud/WK8c) Thera-Py uses all three files provided as the HemOnc CC-BY subset: YYYY-MM-DD.ccby_concepts.tab, YYYY-MM-DD-ccby_rels.tab, and YYYY-MM-DD-ccby_synonyms.tab. Extracted data includes concept identifiers, labels, trade names, aliases, and cross-references. Additionally, the year of approval by the FDA and a listing of indicated diseases and conditions is acquired.

*NCI Thesaurus* is an initiative by the National Cancer Institute to “integrate molecular and clinical cancer-related information within a unified biomedical informatics framework, with controlled terminology as its foundational layer.”[^12^](https://paperpile.com/c/0sK7Ud/eVGl) While its focus is oncology, it has broad coverage of related clinical and scientific knowledge. Thera-Py retrieves therapy identifiers, labels, aliases, and cross-references for all classes that descend from “Pharmacologic Substance” (Code C1909), and all classes that have the semantic type “Pharmacologic Substance” but do not have the semantic type “Retired Concept”. Data is pulled using the NCIt Web Ontology Language distribution (Thesaurus_XX.XX.OWL.zip) provided on the NCI FTP server (<https://evs.nci.nih.gov/ftp1/NCI_Thesaurus/Thesaurus_23.04d.OWL.zip>).

*RxNorm* is a resource provided by the National Library of Medicine to better enable “communicating about clinical drugs and supporting interoperation between drug vocabularies.”[^21^](https://paperpile.com/c/0sK7Ud/XOJB) RxNorm is constructed from a number of source terminologies with varying licensing agreements; Thera-Py uses information from RxNorm that is provided with a UMLS Source Level Restriction value of 0 or 1. Within those restrictions, Thera-Py gathers drug identifiers, aliases, brand and trade names, cross-references, as well as an indication of a drug’s legal prescribable status within the United States. Thera-Py collects this data from the latest available RxNorm release (RxNorm_full_MMDDYYYY.zip), provided on the UMLS downloads page (<https://www.nlm.nih.gov/research/umls/rxnorm/docs/rxnormfiles.html>).

*Wikidata* is an open, collaborative knowledge-base that collects factual claims and descriptions in a Semantic Web model. It is often employed in machine learning and text mining research to provide structured knowledge about a variety of topics. Rather than regular data releases, Wikidata exposes a SPARQL query endpoint for custom requests. Thera-Py requests all instances of the class “Medication” and its subclasses, and retrieves IDs, labels, aliases, and available cross-references for each returned class. It saves all data locally in JSON format (as wikidata_YYYY-MM-DD.json) and loads data from there.
