# Supplementary material for: Normalization of drug and therapeutic concepts with Thera-Py
Source: JAMIA Open. 2023 Nov 8;6(4):ooad093. doi: 10.1093/jamiaopen/ooad093 (PMC10637840; doi:10.1093/jamiaopen/ooad093)
Supplement: ooad093_Supplementary_Data [file ooad093_supplementary_data.zip › TheraPy - Supplemental Figure Legend.docx]

**Supplemental Figure 1. Thera-Py normalizes therapeutic records under merged therapeutic concepts. (A)** Parent node representation used to assign stable concept identifiers for unique therapeutic concepts. 16,069 unique therapeutic concepts were identified from imported identities. Anchor nodes were selected for each concept from the highest priority available source as defined by our internal priority list. Source priority is represented via verticality (Top: RxNorm, Bottom: ChemIDPlus). **(B)** Distribution of the number of records combined under each unique therapeutic concept.

**Supplemental Figure 2. Normalization of therapeutic terminology through Thera-Py.** Schematic for normalization of therapeutic terms via Thera-Py. (1) Raw drug inputs from existing clinical applications are provided to Thera-Py as queries. (2) Thera-Py compares queried terms (submitted via REST or Python API) against stored aliases, trade names, and other therapeutic descriptors to identify the corresponding merged concepts. (3) Thera-Py returns stable merged concepts for downstream usage in clinical applications. If the search term does not exist within the database, no match is returned.
