# Supplementary material for: Normalization of drug and therapeutic concepts with Thera-Py
Source: JAMIA Open. 2023 Nov 8;6(4):ooad093. doi: 10.1093/jamiaopen/ooad093 (PMC10637840; doi:10.1093/jamiaopen/ooad093)
Supplement: ooad093_Supplementary_Data [file ooad093_supplementary_data.zip › TheraPy - Supplemental Tables.docx]

| **Merged Group** | **Records** | **Label** |
| --- | --- | --- |
| rxcui:21245 | 86 | Clobetasol propionate |
| rxcui:104466 | 77 | Tinzaparin sodium |
| rxcui:1545992 | 76 | Doxycycline anhydrous |
| rxcui:203188 | 76 | Methylphenidate hydrochloride |
| rxcui:114477 | 67 | Levetiracetam |
| rxcui:8591 | 63 | Potassium chloride |
| rxcui:2582 | 61 | Clindamycin |
| rxcui:10759 | 61 | Triamcinolone |
| rxcui:1429044 | 57 | Ibuprofen, sodium salt |
| rxcui:281 | 55 | Acyclovir |
| rxcui:203204 | 54 | Bupropion hydrochloride |
| rxcui:7804 | 51 | Oxycodone |
| rxcui:235743 | 50 | Metformin hydrochloride |
| rxcui:24395 | 49 | Estradiol valerate |
| rxcui:8703 | 49 | Fenofibrate |
| rxcui:6922 | 47 | Metronidazole |
| rxcui:203150 | 47 | Cetirizine hydrochloride |
| rxcui:103468 | 45 | Hydrocortisone butyrate |
| rxcui:10379 | 44 | Testosterone |
| rxcui:4462 | 44 | Fluocinonide |
| rxcui:136411 | 44 | Sildenafil |
| rxcui:115713 | 43 | Montelukast sodium |
| rxcui:218337 | 42 | Methadone hydrochloride |
| rxcui:11124 | 42 | Vancomycin |
| rxcui:227224 | 42 | Fluoxetine hydrochloride |

**Supplemental Table 1. Merged therapeutic concepts with the largest number of combined records within Thera-Py.** Merged therapeutic concept groups with the most combined records (top 25 shown).
